# Optimizing Surgical Correction of Symblepharon Secondary to Lower Eyelid Burn Scar

**DOI:** 10.7759/cureus.75933

**Published:** 2024-12-18

**Authors:** Andre Pinto, Manuel Caneira, Francisco Caneira, Diogo Conduto, Catarina Gouveia

**Affiliations:** 1 Plastic and Reconstructive Surgery, Hospital de Santa Maria, Unidade Local de Saúde de Santa Maria (ULSSM), Lisbon, PRT; 2 Plastic and Reconstructive Surgery, Hospital CUF Descobertas, Lisbon, PRT

**Keywords:** buccal mucosal graft, eyelid burn, eyelid reconstruction, lower eyelid retractor, symblepharon

## Abstract

The healing of lower eyelid injuries can lead to anatomical distortion and compromise the function of multiple periocular structures. To restore eyelid function and aesthetics, it is crucial to establish an accurate diagnosis and provide appropriate treatment to the affected structures. The presented case describes the diagnosis and surgical treatment of a lower eyelid symblepharon, which can be easily misdiagnosed as other scarring sequelae.

## Introduction

The eyelids serve as protective and lubricating structures for the ocular globe. The upper eyelid exhibits dynamic movement, while the lower eyelid functions primarily statically. The eyelids are composed of three lamellae: the anterior lamella for external coverage, the middle lamella for structural support, and the posterior lamella for deep support with mucosal lining [1,2]. The treatment of eyelid injuries should aim to restore functional coverage while simultaneously preventing recurrence [3]. This restoration should encompass the different layers of the eyelid [1,3]. Burned lesions can result from both anatomical injuries caused by trauma and from abnormal healing processes characterized by excessive scarring [4]. Symblepharon is a condition that occurs when abnormal healing after injury to the conjunctiva leads to adhesion between the bulbar and palpebral conjunctiva [4].

## Case presentation

**Figure 1:**
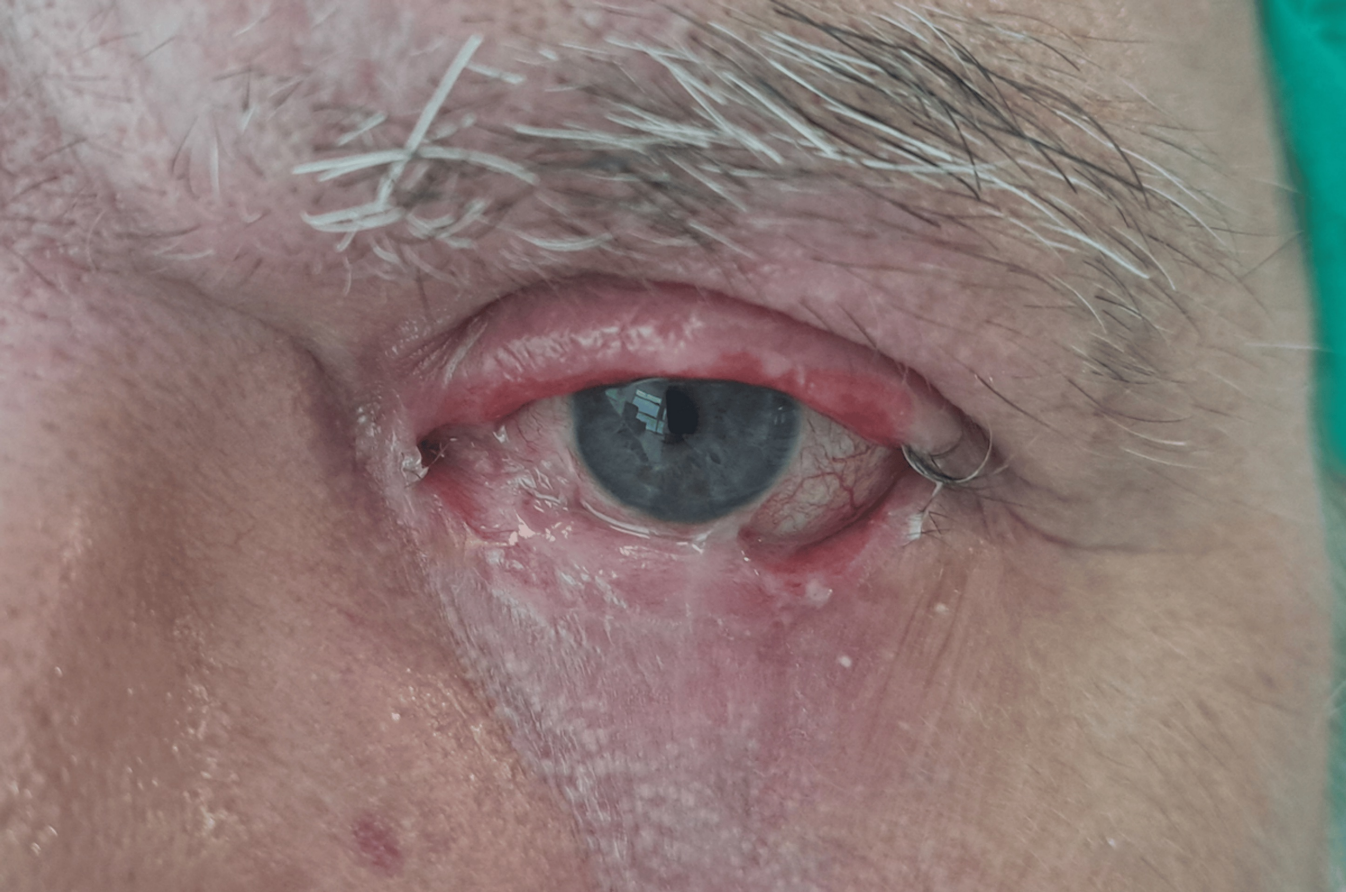
Preoperatory Symblepharon of the left lower eyelid secondary to burn adhesion

A 54-year-old male patient with a history of hypercholesterolemia and hypertension sustained an open-air flash burn while manipulating an explosive plastic device. Initial treatment at a burn unit included topical chloramphenicol (six times daily), dexamethasone (twice daily), and daily frequent lubrication with Davilose® and GenTeal™ Gel, leading to secondary healing. The patient was discharged after two weeks. He presented to our institution five months post-burn discharge for evaluation and management of sequelae, specifically symblepharon, as shown in Figure 1.

The grade of the adhesion resulted in an over 75% loss of inferior fornix depth (Stage 4 Mondino and Brown) with compromised globe coverage, and surgical treatment was required. In the case presented, the anterior and middle portions of the eyelid did not present clinical adhesions nor clinically impair scarring. Lower eyelid skin and tarsal integrity did not result in ectropion or entropion. As such, it was decided not to address the reconstruction of the anterior (outer) or middle lamellae.

**Figure 2:**
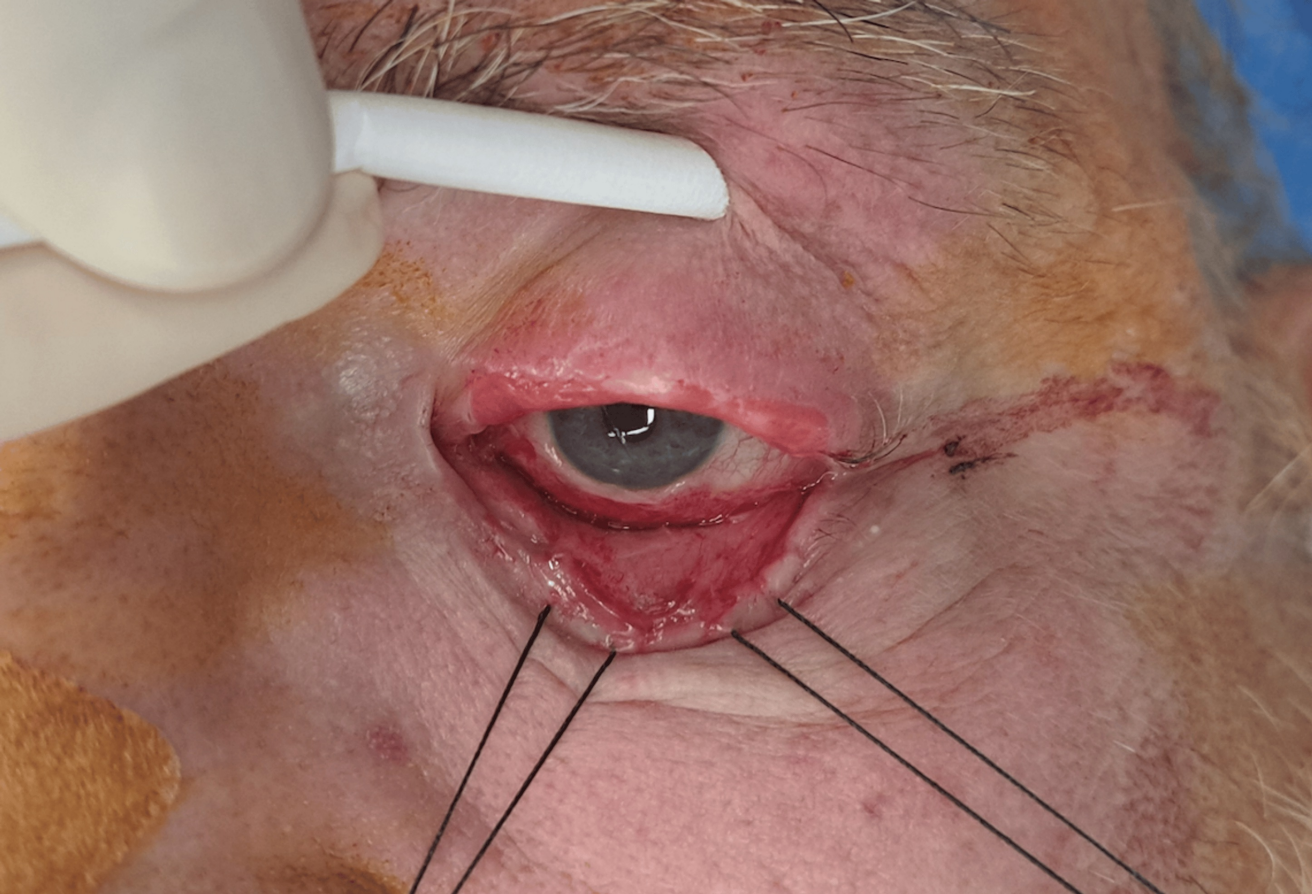
Adhesion release Release and debridement of the lower eyelid scar

Under general anesthesia and optical loupes magnification aid, surgical release of adhesion between the bulbar and palpebral conjunctiva was performed, with care not to allow for globe injury nor desiccation. Measurement after release determined an approximate 2,5 cm x 3 cm palpebral conjunctiva defect, as shown in Figure 2.

**Figure 3:**
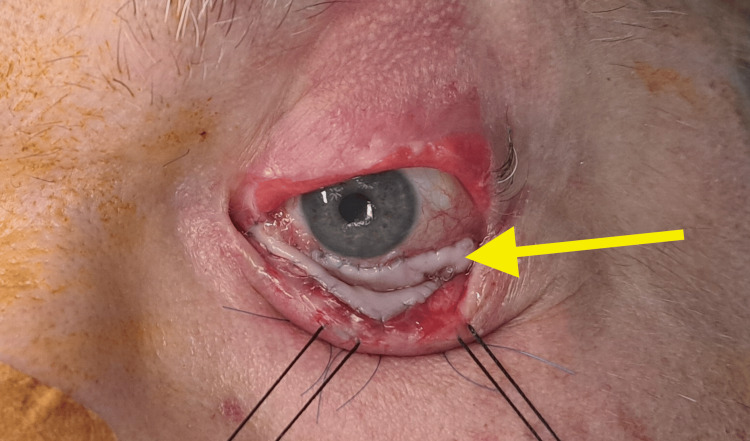
Grafting Bucal mucosa graft with 9-0 suture (arrow)

The lower lip inner mucosal graft was determined to be an adequate donor site allowing for direct suture after harvest. After proper inspection and thinning of the mucosal graft, inset with absorbable suture Vicryl® 9-0 allowed for complete coverage of the inner lower eyelid. For adequate coaptation, three transpalpebral anchorage suture knots were also applied. These anchor points were performed in a transfixed fashion, through the lower palpebral skin and the mucosal fornix, between the ocular and palpebral mucosal reflection point. The inset is illustrated in Figure 3.

**Figure 4:**
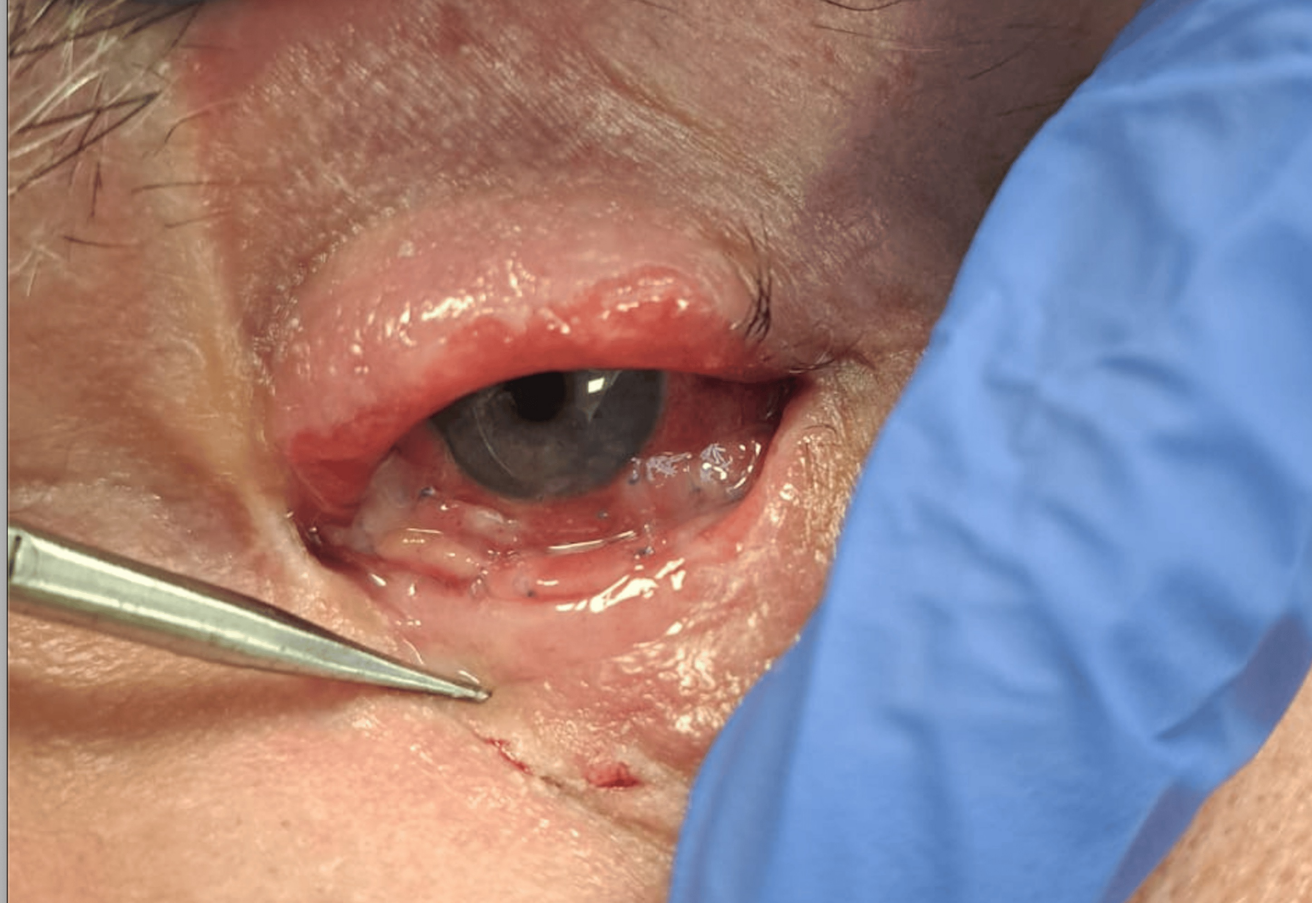
Postop Graft taken on the 10th day after surgery

A conventional simple eye cover bandage was provided, and the patient was discharged for ambulatory follow-up with an ophthalmic drops prescription. Additionally, temporary tarsorrhaphy was applied for one week for optimal graft take, which occurred without intercurrence as shown in Figure 4.

## Discussion

Symblepharon is characterized by the adhesion of the palpebral conjunctiva to the bulbar conjunctiva, often resulting from trauma, chemical burns, or thermal injuries [5]. This condition can lead to impaired eyelid mobility, reduced ocular surface lubrication, and potential vision complications. Other distinct conditions affecting the lower eyelid and ocular surfaces such as ectropion, entropion, or lagophthalmos can mimic symblepharon so proper diagnosis relies on understanding their pathophysiology, clinical features, and differentiators [6,7].

Ectropion corresponds to the outward turning of the eyelid margin with exposure of palpebral conjunctiva but without adhesions. The eyelid everts without ocular surface adherence, typical of symblepharon [8,9]. Entropion in an inward turning of the eyelid margin leads to trichiasis, eyelashes rubbing against the cornea, without adhesions to the conjunctiva [9,10]. Lagophthalmos is the incomplete eyelid closure during blinking or sleep. Unlike the other conditions, lagophthalmos involves dynamic dysfunction leading to exposure-related complications [11]. Table 1 summarizes key differentiators.

**Table 1:** Key differences Key clinical differences between symblepharon and its mimics [7-11]

Feature	Symblepharon	Ectropion	Entropion	Lagophthalmos
Pathophysiology	Conjunctival adhesions	Outward turning of eyelid	Inward turning of eyelid	Incomplete eyelid closure
Adhesions	Yes	No	No	No
Movement restriction	Yes (due to adhesions)	No	No	Yes (impaired closure)
Eyelid position	Normal (adhered to globe)	Everted	Inverted	Partially open
Symptoms	Restricted gaze, dryness	Tearing, irritation	Pain, corneal irritation	Dryness, exposure keratopathy
Causes	Burns, cicatricial disease	Age, scars, facial palsy	Age, scars, spasm	Facial palsy, trauma, surgery
Treatment	Adhesiolysis, mucosal grafts	Horizontal tightening, skin grafts	Retractor reinsertion, scar release	Tarsorrhaphy, gold weight implantation

The surgical correction of symblepharon is particularly challenging in cases involving burn scars, as the underlying fibrosis exacerbates tissue contracture and limits conjunctival mobility [10]. In our case, burn scar release is the cornerstone of symblepharon correction, providing a critical foundation for graft placement. Mucosal graft harvested from the buccal mucosa is highly suitable due to resilience to contraction and structural compatibility with conjunctival tissue [6]. Careful preparation of the recipient bed to ensure graft adherence is also essential [6]. Postoperative care is equally crucial, with strategies such as ocular lubricants to promote healing and minimize complications like graft necrosis or infection [6,7]. Although mucosal grafts are effective, their application requires precise surgical planning and techniques such as loupes-assisted magnification and microsurgery techniques. 

To prevent readhesion of symblepharon, various tissues such as autologous conjunctival grafts, oral mucosa, amniotic membrane transplantation (AMT), and nasal mucosa have been employed to cover the affected areas. In severe bilateral ocular surface disease, AMT loaded with limbal stem cells or allogeneic cultivated limbal epithelial transplantation (CLET) allows to freely address size-related concerns and overcome other donor site limitations [11]. Nevertheless, immune rejection remains a significant challenge, as it can lead to cell death and failure of ocular surface reconstruction [11].

Recurrence of symblepharon remains a concern, particularly in patients with extensive scarring or autoimmune conditions, warranting long-term follow-up [10]. Cheng et al. reported 50% recurrence in 80 eyes treated with CLET [11]. Jain and Rastogi reported recurrence in eight out of 20 patients treated with AMT [12]. The success of surgical treatment relies on the severity of the symblepharon, the level of preoperative inflammation, and the presence of postoperative complications [11].

## Conclusions

Accurate differential diagnosis of eyelid conditions such as symblepharon, entropion, ectropion, and lagophthalmos is essential for condition-specific management, reducing the risk of complications like keratopathy and vision loss. Mucosal grafts are particularly suited for inner eyelid reconstruction due to their low donor site morbidity and favorable integration. Since defect-donor site mismatch was not a concern, autologous mucosal graft was considered the best treatment option against alternatives such as AMT or CLET. Graft take occurred without complications restoring lower eyelid coverage and function. This case highlights the critical importance of understanding the intricate anatomy and function of the eyelid to ensure effective reconstruction.
